# Nondestructive Electrochemical Identification of Lithium Plating in High-Energy Automotive Batteries

**DOI:** 10.1021/acsomega.4c10805

**Published:** 2025-03-25

**Authors:** Syed Muhammad Abbas, Christoph Drießen, Marvin Sprenger, Christian Ellersdorfer, Ilie Hanzu, Gregor Gstrein

**Affiliations:** † Vehicle Safety Institute, Graz University of Technology, Graz 8010, Austria; ‡ Institute for Chemistry and Technology of Materials, Graz University of Technology, Graz 8010, Austria; § Mercedes-Benz AG, HPC X631, Sindelfingen 71059, Germany; ∥ ALISTORE—ERI European Research Institute, CNRS FR3104, Hub de L’Energie, Rue Baudelocque, Amiens F-80039, France

## Abstract

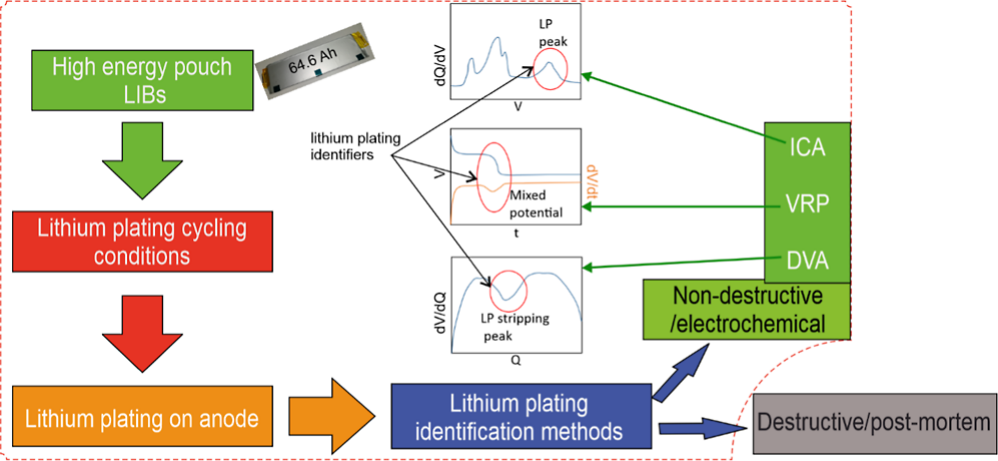

In this study, commercial 64.6 Ah automotive pouch-cell lithium-ion batteries were used to identify lithium plating (LP) by *operando* nondestructive electrochemical methods. These methods rely on the analysis of the cell voltage and cell current data, which is usually acquired by a battery management system (BMS). The cells were cycled under specific cycle-rate and temperature boundary conditions (BCs) in order to induce LP. The selected BCs were 0, 10, and 22 °C as test temperatures in combination with 1, 1.5, 2, and 3 C as C-rates. The chosen electrochemical methods are voltage relaxation profile (VRP), differential voltage analysis (DVA), and incremental capacity analysis (ICA). These methods are applied to different sections of charge/discharge cycle voltage and current data to identify the LP. ICA can be applied to the constant current phase of the charge cycle, VRP can be applied during the relaxation time after the charge cycle has finished, and DVA can be applied to the CC-phase of the discharge cycle. These methods are promising, as they do not require additional equipment for data acquisition, proving feasible for online monitoring by the BMS in an electric vehicle. Our investigation shows that in the case of VRP time derivative analysis, with decreasing temperature and increasing C-rate, the plateau/peak position shifts to the right, indicating increased LP during the charge cycle. During the DVA, the characteristic Li-stripping peak shifted to higher capacity values at lower temperatures and higher C-rates. Surprisingly, at a test temperature of 0 °C, the LP is higher at a rate of 2 C than at a rate of 3 C. This effect is attributed to the higher cell polarization at 3 C than at 2 C during the charge phase. In the ICA, at cycling rates of 1.5 C and above, an additional peak was observed at cell voltages above 4 V. This new peak represents an additional reaction taking place at the anode surface, identified as LP. For 0.5 C, no LP was identified in ICA, and LP was only identified at 0 °C at 1 C, whereas LP was always identified at cycling rates of 2 and 3 C at all test temperatures. The described methods and findings open the path toward advanced online LP identification and eventually state-of-health diagnosis of automotive batteries.

## 1 Introduction

Lithium-ion batteries (lithium-ion batteries (LIBs)) are currently the leading energy storage devices for the automotive application industry, electronic devices, and energy storage systems.^1,2^ However, the performance of LIBs (power and capacity) is typically reduced over their lifetime due to the so-called aging. The phenomenon of aging includes multiple degradation mechanisms, whereby active material (AM) particle cracking and delamination, electrolyte decomposition, solid electrolyte interphase (SEI) growth, lithium plating (LP), and current collector corrosion are the most relevant examples.^3−5^ These aging mechanisms are the effects of the operation of the battery at high or low temperatures, at a high state of charge (SOC) under possible overcharge conditions, at a low SOC under possible overdischarge conditions, at high C-rates, under mechanical stresses, etc.^3−5^ The described aging mechanisms lead to the most relevant degradation modes, namely the loss of lithium inventory (LLI), loss of anode AM (LAM_NE_), and loss of cathode AM (LAM_PE_),^3,4^ which are responsible for the reduction of battery capacity and power.

LP is one of the important aging mechanisms, which leads to LLI. Under normal operation conditions, the lithium cations (Li^+^) are intercalated into the anode materials during the charge cycle, whereas under certain conditions, instead of intercalation, Li^+^ is plated on the anode surface as Li (lithium) metal, in either dendritic or mossy form.^6,7^ The specific boundary conditions (BCs) that induce Li plating are high C-rates, high SOC, low temperature, and high localized pressure^6−9^ during cycling. LP can be further divided into two categories, namely, reversible and irreversible LP. Reversible LP can be stripped back as Li^+^ into the electrolyte during relaxation after charging or during the discharge cycle. Additionally, an intercalation into the anode occurs during relaxation time.^6,7^ Irreversible Li plating occurs when the plated dendritic Li is exposed to the electrolyte. During discharge, the stripping of Li metal starts on the interface between the anode and plated dendritic Li.^7^ Thereby, the top of plated Li can break off and lose electric contact with the electrode, and the formerly inner surface is exposed to the electrolyte. As soon as new SEI forms around this metallic Li, it cannot contribute to further intercalation and deintercalation reactions^6,7,10,11^ as SEI is electrically nonconductive.^12,13^ Thus, since the contact with the anode is lost, Li in this state is typically referred to as ‘dead Li’.^7^

Multiple methods to identify LP are reported in the literature^7,14,15^ and summarized by Paul et al.^16^ For the present study, nondestructive electrochemical methods were selected to investigate and identify LP, namely voltage relaxation profile (VRP),^17−20^ differential voltage analysis (DVA),^21−25^ and incremental capacity analysis (ICA).^19,26−28^ VRP and DVA curves identify the Li-stripping reaction, whereas ICA curves identify LP formation. VRP shows lithium stripping as a plateau, DVA shows lithium stripping as a typical peak at a specific capacity value, and ICA represents LP as a reaction peak at distinct voltage.^6−9^ These nondestructive methods can be applied to voltage and current data of cells in use to identify LP without additional equipment needed, which makes them eligible for low-effort and low-complexity monitoring. Other reported methods such as EIS, thickness measurement, etc. require additional equipment such as a potentiostat and a displacement sensor, respectively, limiting their *operando* application.^7,14,15^

So far, the selected methods were used to investigate LP in experimental cells^19,20^ and low-energy commercial cells.^17,18,21^ The cell chemistries analyzed were graphite/Li,^20^ graphite/NMC111,^18^ graphite/NMC532,^19^ and graphite/LFP.^21^ A gap in the published literature was identified, as there is a lack of information regarding these selected methods used for investigating LP in commercial high-energy cells for automotive applications. It is expected that due to the large size and high number of layers in such cells, gradients of temperature^29^ or Li-ion concentration^30,31^ gradients can develop. These factors may lead to a different behavior compared to that of the available published data. Furthermore, the influence of silicon (Si) as a component of anode AM, which is typically added to increase the anode energy density^32^ and fast charging capability^33^ (gravimetric capacity of Si = 4200 mA h g^–1^^34^) was not yet reported for experiments carried out under LP conditions. Although the electrochemical properties of graphite-SiO_*x*_ are investigated and discussed already in the literature.^35,36^

One aim of this study is the identification of LP and evaluation of the selected methods to identify LP in commercial high-energy LIBs. Such methods are useful for online monitoring by the battery management system (BMS) due to their noninvasive nature. In addition, the correlation of the selected methods with each other and their relative accuracy will also be considered. Thereby, a focus is put on how the amount of Li-stripping (reversible LP) as a result of DVA and VRP correlates to the formation LP, as found in ICA. In addition, the combination of all three methods for LP identification and the complementary information they offer is first reported in this comprehensive study.

## 2 Methodology

VRP is an *operando* method to detect reversible LP in LIBs; it is a nondestructive technique measuring the open circuit voltage during the relaxation time after charging.^37^ A plateau in voltage during the relaxation time, caused by a mixed potential, is the indication of LP. This plateau corresponds to Li stripping or Li intercalation into the anode.^20,37^ In the absence of LP, no voltage plateau is observed during relaxation. The time derivative of VRP plotted with respect to time gives a peak instead of the plateau, and the position of the peak is an indication of the amount of LP.^37^ The steepest point of the VRP curve corresponds to the minima in the time derivative. After this point, the rate of stripping decreases and voltage starts to normalize.^18^ The peak position on the *x*-axis represents the duration and thereby also indirectly the amount of Li-stripping.^18,19,38^ For the detection of LP with VRP and the corresponding time derivative, the amount of LP should be more than 1% of the graphite capacity.^16,20^

In contrast to VRP, DVA is calculated for the discharge cycle constant current (CC) phase. DVA gives a characteristic peak in the initial phase of the curve, whereby the peak position on the capacity *x*-axis corresponds to the amount of stripped or reversible LP.^16,21^ A relaxation time after a charge cycle influences the DVA characteristic peak signal; a longer relaxation time leads to a weak DVA peak signal. As stripping or intercalation also occurs during relaxation time, that amount of Li-stripping signal is lost and not recorded during the discharge cycle Li-stripping reaction. DVA identifies Li-stripping if the plated amount of LP in the charge cycle is 1–5% of graphite capacity.^16,39^ Additionally, the characteristic Li-stripping peak is observed when the cell is discharged from a high SOC, as LP is more likely to have occurred during charging.^14,21^ However, discharging from a low SOC fails to exhibit a Li-stripping peak, regardless of whether the cell was charged under LP-inducing conditions.

ICA or dQ/dV is the inverse of the DVA and is applied to the charge cycle CC phase. It is a global, nondestructive, *operando* method which enables the quantification and identification of the degradation modes LLI and LAM.^19,26−28^ This method provides quantification of degradation modes, as it represents a change in capacity with cell voltage. Each electrode material, such as NMC, NCA, graphite, etc., has specific characteristic curves where the peaks represent reactions such as lithiation and cathode phase change at specific voltages.^26^ In commercial LIBs, this characteristic curve is a blend of anode and cathode materials and is influenced by the cell chemistry.^26^ Lithiation and NMC phase change peaks in ICA curves occur at cell voltages below 4 V for graphite-NMC chemistry,^26^ whereas an LP peak is formed at higher cell voltages of 4 V and above.^19^

The selected electrochemical methods, VRP, DVA, and ICA will be used to identify characteristic electrochemical peaks/signals representing LP. Identification of LP in the VRP time derivative and DVA will be confirmed using ICA. A qualitative analysis will be carried out to identify LP in the investigated commercial high-energy LIBs. Further, the correlation of each method will be analyzed for different LP BCs.

## 3 Experimental Section

This study was carried out using commercial high-energy pouch LIBs used in a current electric vehicle. The specifications are described in Table 1.

**Table 1:** Cell Specifications

cell type	large format pouch cell
cell structure	layered
chemistry	NMC712/graphite + SiO_x_
body dimensions	354 × 101 × 11.4 mm
pouch dimensions (cooling area)	318 × 100 mm
tabs dimensions	30 × 7–10 × 0.6 mm
weight	896 g
capacity	64.6 Ah
nominal voltage	3.665 V
maximum voltage	4.25 V
minimum voltage	2.8 V
volumetric energy density (C/3; 25 °C)	648 W h/L
gravimetric energy density (C/3; 25 °C)	263 W h/kg

The cells were cycled using a CC–constant voltage (CC–CV) charge–discharge protocol, with a cutoff current limit value in the CV phase set to C/10. Before the test, the cells were discharged to SOC 0% and rested for 15 min, followed by the first cycle charging to SOC 90%. The SOC was estimated based on coulomb counting, and a time limit was set to end the charge cycle when the estimated time to achieve SOC 90% is reached. This time limit changed with test C-rates, as a higher at C-rate time to reach SOC 90% is low. The charge cycle was followed by a relaxation time of 1.5 h, subsequently followed by a discharge cycle to SOC 0% with CC–CV. This procedure was followed by a second charge–discharge cycle, however, without relaxation time. The respective details are shown in Figure S3. The VRP and the time derivative were calculated during the relaxation time after the first cycle charge. The second cycle discharge curve was used to conduct the DVA (see Figure S3). The ICA curves are derived based on the charge cycles (see Figure S3). The BCs during cycling were selected based on conditions inducing LP and are summarized in Table 2.

**Table 2:** Cell Cycling BCs Used

boundary conditions	
temperature (°C)	0, 10, and 22 °C(nominal ≈ −30 to +60 °C)
C-rate	1, 1.5, 2 and 3 C (nominal ∼0.77 C)
pretension (N)	300 N (nominal)

The cells were cycled, as described above (see the detailed information in Figure S3), at each test temperature and for all indicated C-rates, as indicated in Table 2. The electrical cycling of the cells as well as the data acquisition and recording of measurement data was done by a battery testing, monitoring, and cycling unit built in-house at VSI TU Graz (see Figure S1). This unit was designed by the institute and has a specially programmed cycling software, enabling it to cycle cells with currents ranging between 0 and 340 A, with a data acquisition frequency of a maximum of 2000 Hz. The data acquisition frequency used during the cycling was defined as 10 Hz, and data analysis was performed using DIAdem (National instruments).

The cell was pretensioned with a force of 300 N evenly distributed over the entire cell surface. This reflects the nominal pretension of the selected cell in normal operation. During cycling, the cells were actively cooled using aluminum plates with water-cooled Peltier elements to achieve constant test temperatures. The temperatures were measured on each Peltier element with the help of PT100 sensors with an accuracy of 0.1 °C. The difference in test and achieved temperature was approximately 0.5–1 °C. The change in temperature while cycling was recorded to be 0.1–0.8 °C. Detailed information on the test setup is included in Figure S2, and an overview of the achieved test temperatures while cycling in comparison to the respective target temperatures is summarized in Figure S4.

As a starting point, reference cycles were performed for 0.5 C at 0 and 22 °C to evaluate the electrochemical behavior of fresh cells. Test cycles within the BCs described in Table 2 were carried out and compared to the respective reference cycles in order to identify changes in cell behavior.

## 4 Results

**Figure 1:**
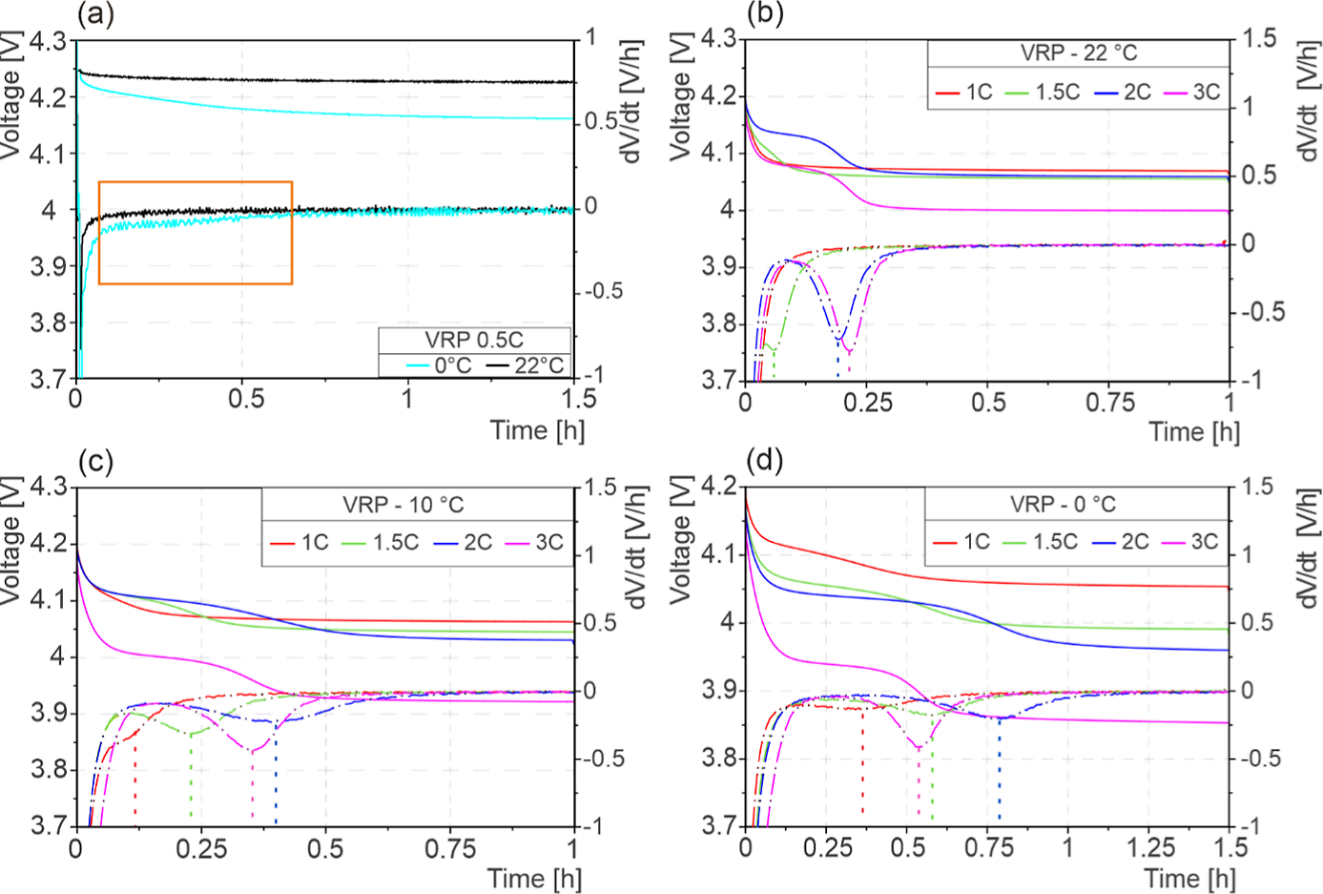
VRP and time derivative d*V*/d*t* of (a) reference profiles at 0.5 C, (b) 22 °C, (c) 10 °C, and (d) 0 °C. Comparison of change in VRP w.r.t. C-rate and temperature.

The VRP and corresponding time derivative (d*V*/d*t*) of the cycled cells are presented in Figure 1. The reference VRP and the corresponding time derivative curves of the cell cycled at 0.5 C with indicated test temperatures are presented in Figure 1a. At 22 °C, no VRP plateau or peak of the respective time derivative are observed. At 0 °C, a slight change in slope was detected in VRP with no significant peak observed in the derivative curve. This retarded relaxation can be attributed to the effect of the low temperature on the cell behavior and the resulting slow dissipation of concentration gradients in the electrolyte within the cell. Figure 1b shows the VRP at 22 °C. No LP is observed at lower C-rates (≤1 C); during cycling at higher temperatures, the LP reaction rate is low as compared to the Li-stripping reaction.^9,40^ For higher C-rates (≥ 1.5C) at 22 °C, voltage plateaus and respective time derivative peaks can be observed, as shown in Figure 1b.

Figure 1c,d shows the VRP and the time derivate at 10 and 0 °C, respectively. Clear VRP plateaus and time derivative peaks were observed for all C-rates at these test temperatures. The time derivative peak minima shift to higher time values for all C-rates at lower test temperatures. This indicates a higher amount of Li-stripping reaction, which corresponds to increased LP during the charge cycle. It was observed that cell voltages relaxed to lower voltages with increasing C-rates and decreasing temperatures, which can be attributed to the rising cell polarization^15^ under such conditions. With increasing C-rate, the cell polarization also increases, which is evident from the relaxation voltage value (Figure 1) and the charge cycle data (Figure S5).

**Figure 2:**
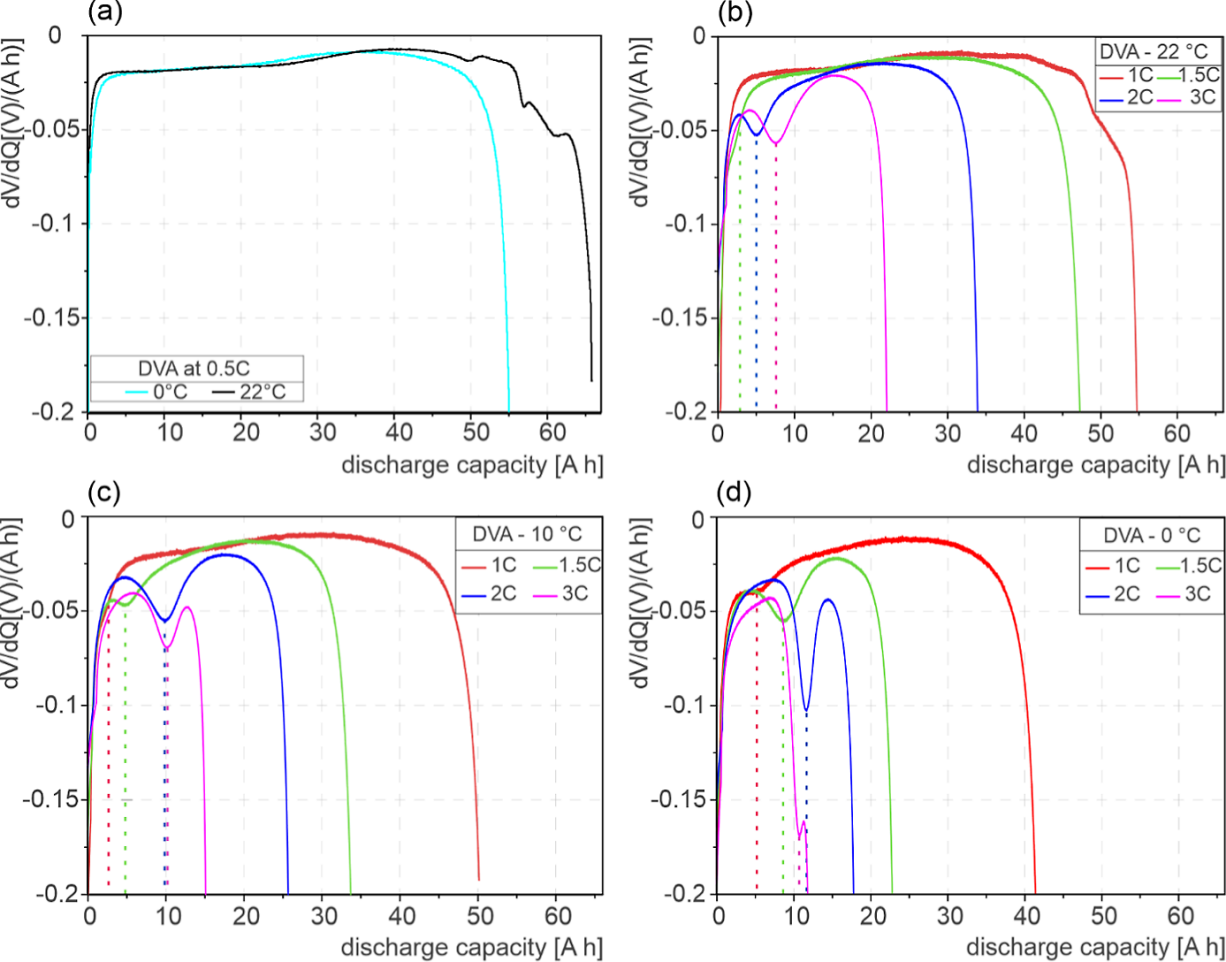
DVA curves showing the influence of C-rate and temperature on Li-stripping. (a) Reference curves at 0.5 C, (b) at 22 °C, (c) at 10 °C, and (d) at 0 °C.

Figure 2 shows the comparison of the DVA for the discharge cycle (CC-phase) of the cells cycled at the indicated BCs. The reference DVA curves in Figure 2a show no Li-stripping peak for 0.5 C.^21−23,41^ However, characteristic peaks representing reactions on the anode and cathode were observed at the end of the discharge cycle above 40 Ah at 22 °C,^21,22,24^ whereas for 0 °C, those peaks cannot be detected. The DVA curves at 22 °C for higher C-rates are shown in Figure 2b. For 1 C, no Li-stripping peak was observed, whereas Li-stripping peaks are observed for 1.5, 2, and 3 C. For 1 C, graphite^21,22,24^ and NMC^23^ phase changes are observed at discharge capacities above 30 Ah; however, they are not as distinct when compared to peaks observed at 22 °C in Figure 2a. In Figure 2c,d, Li-stripping DVA peaks are observed for all C-rates at low temperatures of 0 and 10 °C. The DVA peaks are shifted to higher cell capacities with a decrease in temperature. The effect of cell polarization during charging at higher C-rates is also observed,^21^ which results a lower amount of LP in the charge cycle, especially for 3 C (see Table S10). The DVA of the discharge cycle after relaxation time showed a significantly reduced strength of the characteristic Li-stripping signal, independent from temperature and C-rate during charging; see Figure S6.

**Figure 3:**
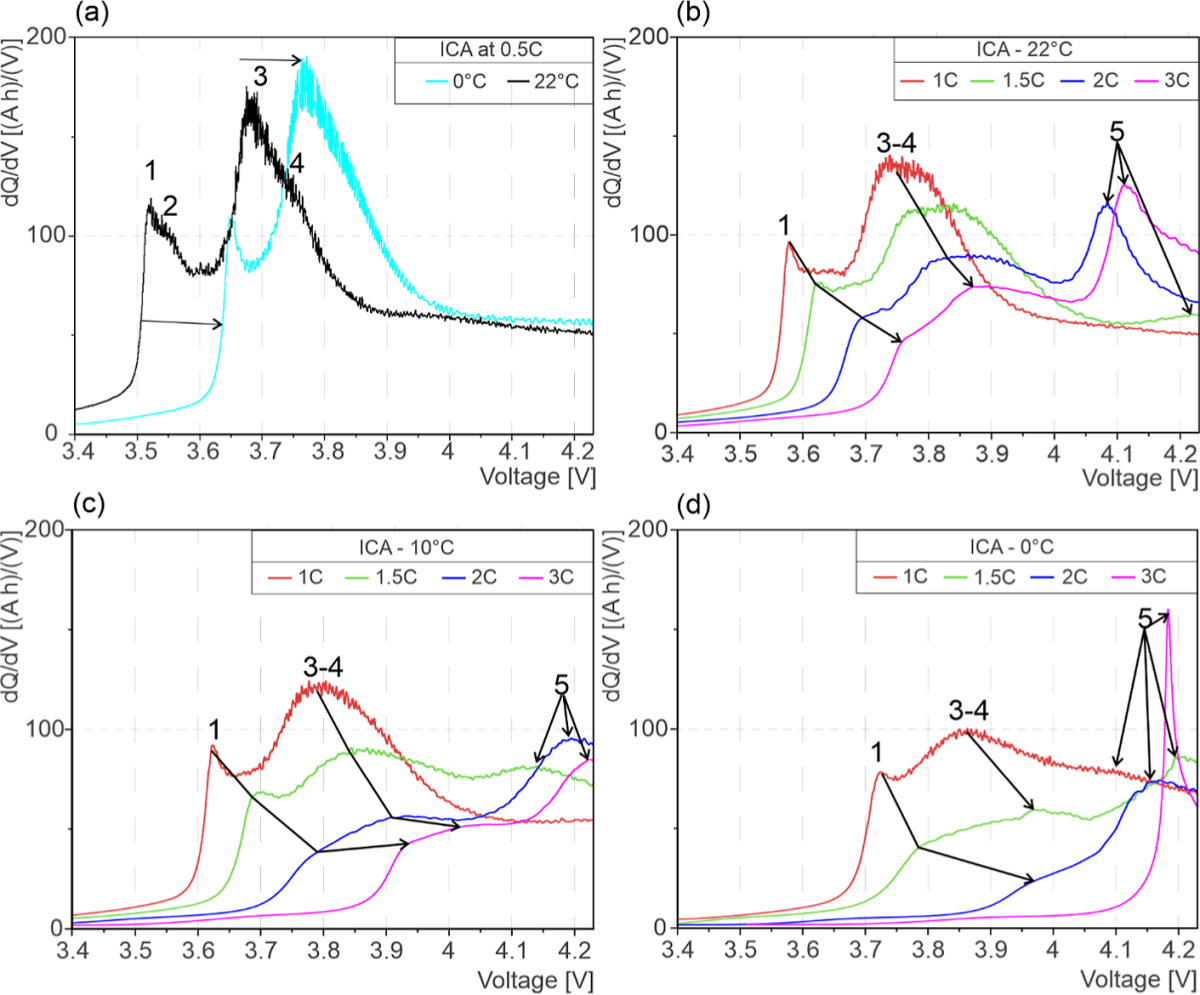
ICA comparison between the reference curve and charge curves under LP BCs showing the influence of temperature and C-rate. (a) Reference ICA curves at 0.5 C, (b) 22 °C, (c) 10 °C, and (d) 0 °C.

In Figure 3, the ICA curves of the charge cycle CC-phase are presented. In the reference ICA curves, as shown in Figure 3a, four different peaks are observed at approximately 3.52, 3.55, 3.68, and 3.75 V, respectively. Peaks 1 and 2 are associated with Li intercalation into graphite-SiO_*x*_,^25,26^ while peaks 3 and 4 are associated with the NMC phase change and NMC solid solution, respectively.^25^ At 0 °C, all peaks are shifted to higher cell voltages, and peak 2 is not observed (see Figure 3a). For test temperature 22 °C, shown in Figure 3b, peak 2 disappears, and no LP peak is observed for 1 C, whereas for higher C-rates, an additional peak 5 (see Figure 3) is observed at cell voltages above 4 V. This new peak 5 represents the LP reaction.^19^ As can be seen on the ICA curves shown in Figure 3c at 10 °C, the intensities of all peaks are reduced for all C-rates. For 1 C, peak 5 was not observed, whereas for 1.5 C, the intensity of peak 5 increased, and the LP reaction occurred at relatively lower voltage compared to 22 °C. In Figure 3c, for 2 C and 3 C, peak 5 was observed at higher cell voltage. Figure 3d shows the ICA curves at 0 °C. A weak LP peak 5 is observed for 1 C, whereas peak 5 is shifted to higher cell voltages for 1.5 C, as compared with Figure 3c. In Figure 3d, for 2 C and 3 C, peak 5 occurred at a relatively lower voltage as compared with Figure 3c. The behavior of the 3 C curve in Figure 3d can be attributed to an extremely short CC phase due to cell polarization at 0 °C, and only the formation of LP is observed. The DVA and ICA peaks associated with SiO_*x*_ lithiation/delithiation^35^ are not visible due to the convoluted nature of the curves.^35,36^

## 5 Discussion

The reference VRP curves show no LP at 0.5 C, as can be seen in Figure 1a. During the charge cycle, no LP occurred for any of the tested temperatures. No LP-indicating plateaus in the VRP and no peaks in its corresponding derivative are observed.^17^ With increasing C-rate, a plateau was formed. Both higher C-rates and lower temperatures showed an increase in the plateau length and shifted time derivative peaks toward the right on the time *x*-axis, see Figure 1b,c. Comparing the data in Figure 1a–d, it was observed that LP does not occur at 0. 5 C, whereas at higher C-rates, LP occurred in the cells and could be identified with VRP and the VRP time derivative. LP occurrence was enhanced by decreasing the temperature and increasing the C-rate. The effect of increasing the C-rate translates into a shift of the time derivative peak to higher values on the *x*-axis at the same temperature.

Comparing the discharge cycle DVA curves in Figure 2a–d, it can be observed that with increasing C-rate, the characteristic Li-stripping peak shifts to a higher capacity on the *x*-axis. Sharp DVA Li-stripping peaks, as the indicator for increased LP, were observed at high C-rates. The influence of temperature on the DVA discharge curves can also be observed, see Figure 2a–d. With decreasing temperature, the characteristic Li-stripping peak moves to higher capacity, representing an increase in Li-plating for C-rates of 1 C and above. The Li-stripping peaks become sharp at lower temperatures when compared to LP peaks at higher temperatures. Sharp DVA peaks were observed for 1.5, 2, and 3 C with a decrease in temperature, as seen in Figure 2b–d. The effect of cell polarization during charging at a low temperature is also evident. The charge capacity is 34.3 Ah at a 3 C in comparison to 40.6 Ah at 2 C, leading to a reduced amount of Li stripping compared to 2 C (see Figure 2d).

In ICA, no LP was observed at 0.5 C, not even at 0 °C, as shown in Figure 3a. However, LP peaks are observed for C-rates of 1.5 and above at all test temperatures, whereas it was only observed at 0 °C for 1 C. The Li-intercalation peaks 1 and 2 shifted monotonically to higher cell voltage with a decrease in temperature due to increased cell polarization, as seen in Figure 3a–d. This observation was also found true for an increasing C-rate at the same temperatures. The shift of the intercalation peaks 1 to 4 to higher voltages with a decrease in temperature and an increase in C-rate is associated with the cell polarization, which depends on charge transfer kinetics, ohmic drops, and mass transport phenomena.^15^ The shift in the voltage of the Li-plating peak is not monotonic when compared with Li-intercalation peaks, as can be observed in Figure 3b–d. At the same temperature, broader peaks were observed with an increase in C-rate. The broadening of Li-intercalation peaks 1 and 2 as well as NMC phase change peaks 3 and 4 with the increase in C-rate is attributed to the variation in the voltage plateau, associated with intercalation peaks. The width of these peaks is influenced by interface kinetics and heterogeneity in local SOC of the electrode surfaces.^42^

In ICA, LP is identified during the charge cycle as a distinct reaction peak above 4 V. This peak is observed for C-rates of 1.5 C, 2 C, and 3 C at all test temperatures. For 1 C, this peak was only observed at 0 °C, whereas for 0.5 C, it was not even at that low temperature. The LP peak identification in ICA correlates to VRP, where a clear plateau and peaks in time derivative were also identified for high C-rates (1.5 C, 2 C, and 3 C). The VRP plateau and time derivative peak were not observed for 0.5 C. At 0 °C, a prominent VRP plateau and time derivative peak are observed for 1C, which is fully correlating to ICA.

By means of DVA, characteristic LP peaks could be identified at C-rates of 1.5 C, 2 C, and 3 C at all test temperatures. For 0.5 C, no LP peaks were observed; for 1 C, the characteristic DVA peak was observed only at 0 °C, which is again in full agreement with ICA, even though the discharge phase is analyzed. The findings of all the selected nondestructive electrochemical methods are in good accordance with each other. LP identified in ICA correlates with the characteristic Li-stripping plateaus and peaks identified in VRP and DVA. The ICA identifies LP in the charge cycle as the distinct peak at cell voltages above 4 V, whereas DVA identifies the stripping back of the plated Li metal as a characteristic peak on the capacity *x*-axis. During relaxation, the stripping back of plated Li metal is identified by the VRP plateau and the corresponding time derivative peak.

The combination of ICA and DVA or VRP, in principle, enables an estimation of irreversible LP, which cannot be directly detected. Thereby, DVA seems to be advantageous since the location of the LP peak directly indicates the discharge capacity from metallic Li, stripped back to the electrolyte. For VRP, the length of the plateau or the position of the time derivative peak represents the duration of the stripping phase, which, however, cannot directly be translated into a corresponding capacity. The capacities stored under LP peak 5 in ICA are higher than the DVA LP stripping capacity, Tables S7–S9; this would suggest an irreversible capacity loss as a result of irreversible LP. In contrast, the capacity loss per cycle is less than the difference between the ICA peak 5 capacity and the DVA LP stripping peak, Table S7–S9. This is a consequence of normal cell reactions responsible for lithium intercalation that occur in parallel with the LP reaction. Thus, both cell reactions contribute to peak 5 capacity. It is not possible to differentiate between the capacity gained by these reactions under peak 5 and the ratio of capacity contribution varying with C-rate. Additionally, the diffusion of lithium in graphite is influenced by temperature, resulting in higher LP as evident by the data. Hence, for simplification, peak 5 capacity as a whole is assumed as LP capacity.

**Figure 4:**
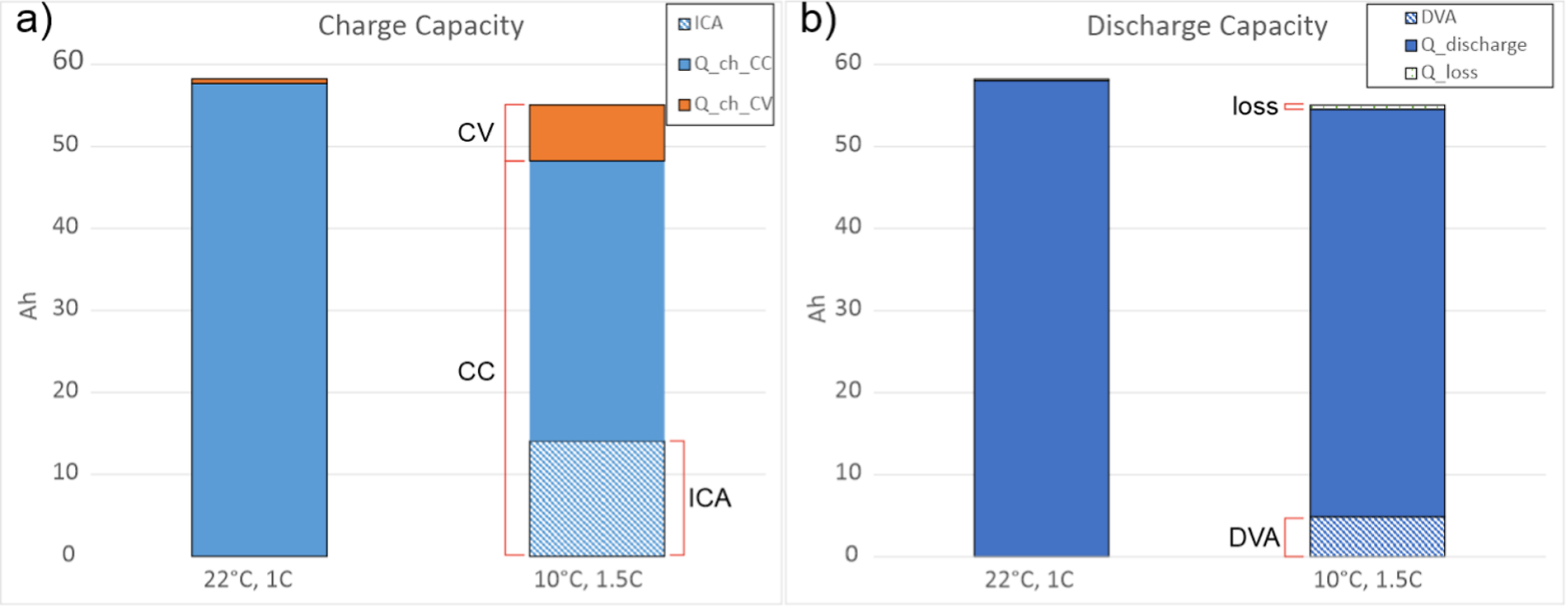
Illustration of cycle capacities at BC: (a) charge cycle and (b) discharge cycle.

The time required for the C-rate to drop below 1 C during the charge CV phase is particularly critical at C-rates of 1.5 C and above. As from the ICA and DVA, it is demonstrated that C-rates higher than 1C show LP. Therefore, it can be safely assumed that LP continues on the anode surface throughout the CV phase as long as the charging current remains above 1C. In Tables S7–S9, the cycle data at the corresponding C-rate is represented at test temperatures of 22, 10, and 0 °C, respectively. It can be observed that while charging, the cells undergo charge CV phase, and this phase is longer for high C rates and low temperatures, indicating cell polarization during charging. The amount of capacity stored during the CV-phase increases with higher C-rates and lower test temperatures. If the difference between ICA peak 5 capacity in Figure 4a and DVA capacity in Figure 4b is assumed to be irreversible LP, then the end cycle capacity loss should be higher. In contrast, the end cycle capacity loss is lower (also see Tables S7 and S9), clearly supporting the argument that both LP and intercalation reactions contribute to ICA peak 5 capacity. Figure 4 illustrates an example of the capacities described in Tables S7–S9. For simplicity of calculations, peak 5 is assumed only as the LP capacity and is calculated by eq 1 and presented in Table S10. Area under peak 5 is estimated as capacity stored as LP during the CC phase (see Figure S12), as LP during the CV phase cannot be quantified. Relative reversible capacity during the discharge phase is calculated using eq 2, and the values are presented in Table S11. These values show reversible LP in DVA as the percentage of LP in ICA peak 5. It can be seen in Table S9 at 0 °C, for 2 C and 3 C, that the DVA LP stripping reversible capacity is higher than the LP peak 5 capacity, supporting the argument that LP occurs during the charge CV phase when the charging current is higher than the LP threshold current, which was determined to be 1 C in this study. Similarly, the relative reversible capacity calculated from eq 2, for 2 C and 3 C at 0 °C, appears higher than 100% (see Table S11). This fact is also attributed to the unaccounted LP formation in the charge CV phase, which increases the LP quantity and results in higher DVA Li-stripping capacity. Since this extra LP occurs in the CV phase, it is not quantified in the ICA peak5 LP capacity.
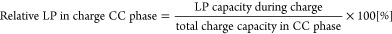
1
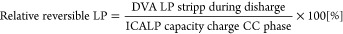
2

While we have presented key features of various nondestructive methods, there are also a series of limitations and shortcomings that must be taken into consideration for an accurate analysis.

•First and foremost, the evaluated methods are based on nondestructive electrochemical measurements and therefore cannot provide physical direct proof of LP. As a next step, destructive post-mortem analysis would be required to confirm the formation of LP. Also, the term nondestructive must be taken with a grain of salt: although the cell case integrity is preserved, once the LP is proven, the cell has already undergone an irreversible capacity loss by losing some of its lithium inventory. While the degradation of the battery state-of-health (SoH) is certain in such an event, LP is not the only cause for the degradation of the cell SoH.
- Further, the estimation of capacity value related to LP formation, as the area under the ICA curve, is complex and a possible source of errors, as the width of a peak is not clearly defined. In order to address this issue, a study on influencing factors and response characteristics should be carried out. In addition, the factors influencing the reversible and irreversible parts of LP are poorly understood. Indeed, little is known, e.g., on how the particular cell chemistry influences LP.
- Finally, from an application point of view, the methods (except for VRP) are only assessed in CC charge/discharge. Thereof, the run of such an analysis as an online-monitoring tool is considered to be challenging. No literature was found on the analysis of ICA, DVA, and VRP based on transient driving data. Yet, our findings show that identification of LP from real-life data would be feasible, provided that both a suitable cell characteristic (experimental) database and a matched data acquisition protocol are implemented, albeit the available data processing power of the current BMS remains hitherto very limited.

## 6 Conclusions

In this work, we have experimentally demonstrated that the three selected electrochemical methods are not limited to experimental or small lithium-ion batteries (LIBs) but can be applied to identify LP also in high-energy commercial LIBs. All three methods have shown clear signs of LP under the respective cycling BCs.

Furthermore, there is a good correlation between the methods, even though each approach analyzes a different phase of the charge–discharge cycle. ICA describes and identifies total LP in the charge cycle, whereas VRP and DVA indicate reversible LP during relaxation time and discharge cycle, respectively. Based on the conducted experiments, the effect of changed cycling BCs on the respective analysis results could also be shown. Thus, in the typical range of operation, the temperature has a more pronounced influence on the risk of LP as compared to the C-Rate. Trends in VRP peaks were observed to be similar to DVA; however, VRP seems to be less straightforward in the further processing of the analysis result. A promising approach to quantifying irreversible LP as the difference between total LP and reversible LP was proposed, although this would require further investigation.

Interestingly, the cycling BCs under which LP was formed and detected seem to be relatively close to nominal operation conditions, as LP was identified under 10 °C at 1.5 C. These BCs can be achieved during normal operation, such as fast charging in a colder environment, especially in winter. Therefore, the importance of proper thermal management of the LIB is clearly emphasized, as is also the pending risk for this type of degradation in the case of a battery thermal management system malfunction.

The analyzed cell featured a different chemistry compared to what was already published in former studies on LP identification. Nevertheless, we were able to verify and confirm that there was a good correlation between different methods used. Thus, our study may constitute the foundation of a scientific and industrial development of advanced *operando* cell SoH diagnosis tools.

## Supporting Information Available

Additional experimental details, results, and photographs of the experimental methods (PDF)

## Abbreviations

LIB: lithium ion battery
LP: lithium plating
VRP: voltage relaxation profile
DVA: differential voltage analysis
ICA: incremental capacity analysis
CC: constant current
CV: constant voltage
C-rate/C: charging rate
BCs: boundary conditionsReferences
