# Supplementary material for: Nondestructive Electrochemical Identification of Lithium Plating in High-Energy Automotive Batteries
Source: ACS Omega. 2025 Mar 25;10(13):13209–17. doi: 10.1021/acsomega.4c10805 (PMC11983349; doi:10.1021/acsomega.4c10805)
Supplement: Supplementary file 1 — ao4c10805_si_001.pdf [file ao4c10805_si_001.pdf]

Supporting information

Non-destructive electrochemical identification of lithium plating in high energy automotive batteries

Syed Muhammad Abbas[1], Christoph Drießen[1], Marvin Sprenger[3], Christian Ellersdorfer[1], Ilie Hanzu[2] and Gregor Gstrein[1].*

1 Vehicle Safety Institute, Graz University of Technology, 8010, Graz, Austria.

2 Institute for Chemistry and Technology of Materials, Graz University of Technology, 8010, Graz, Austria.

3 Mercedes-Benz AG, HPC X631, 71059 Sindelfingen, Germany.

4 ALISTORE – ERI European Research Institute, CNRS FR3104, Hub de l'Energie, Rue Baudelocque, F-80039 Amiens, France

* Email – s.m.abbas@tugraz.at

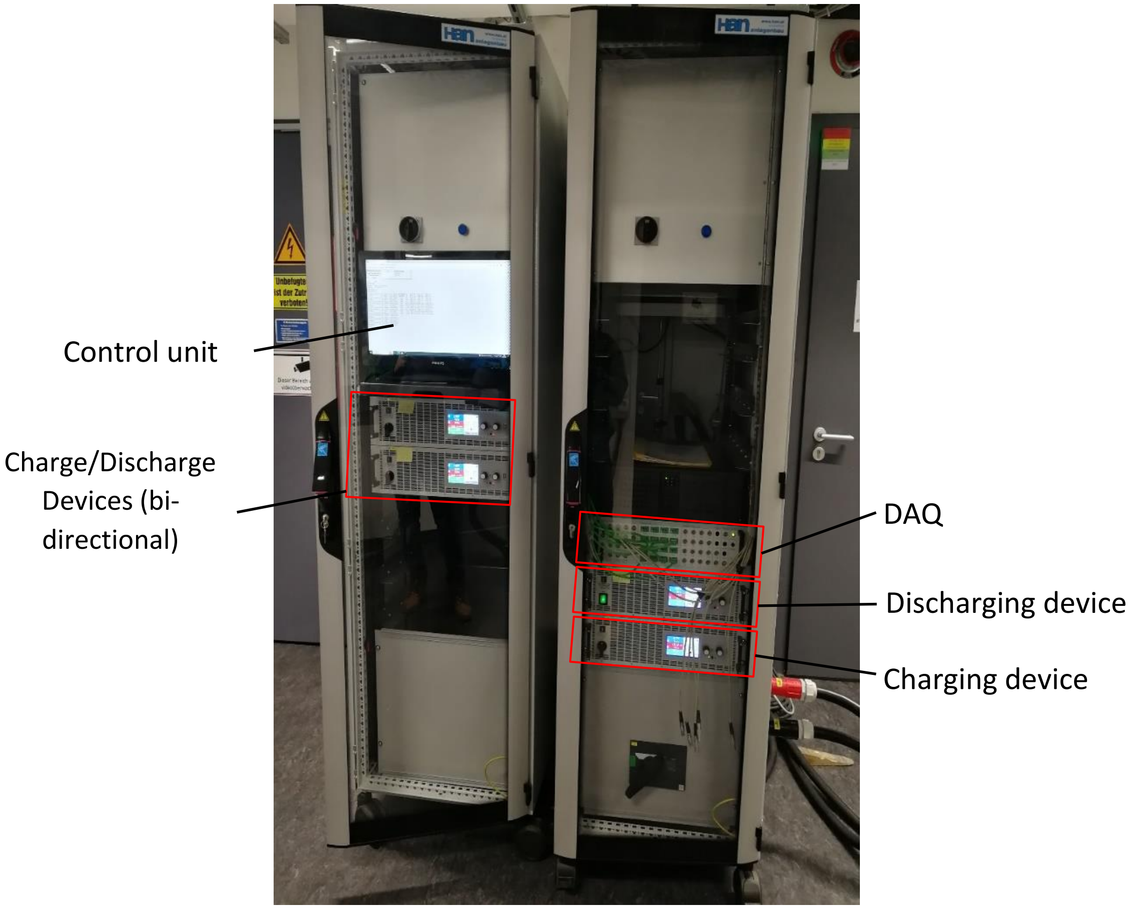

S 1. Battery testing, monitoring and cycling unit.

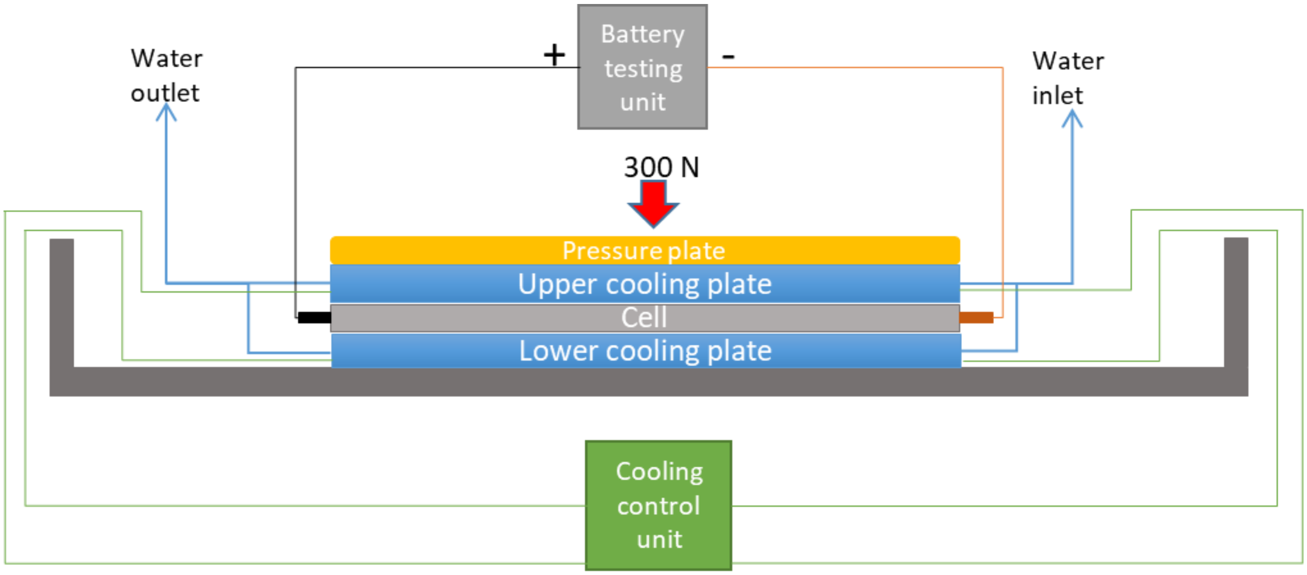

S 2. Test setup representing battery testing unit, cell mounting and active cooling device.

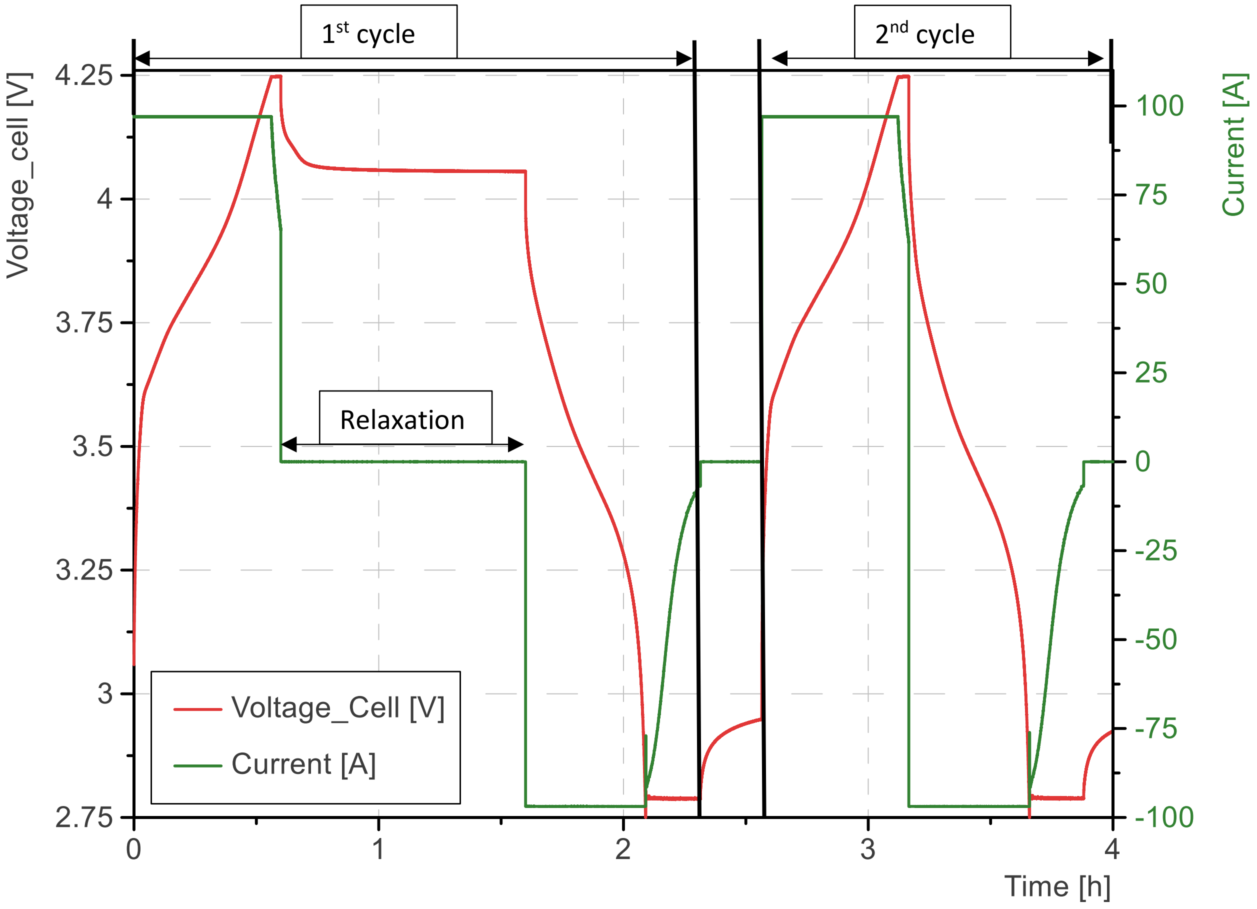

S 3. Implemented charge discharge cycles, indicated cycles and relaxation time.

	Boundary Conditions					
			C-rate			
**Temperature**			**1 C**	**1.5 C**	**2 C**	**3 C**
	**22 °C**	**Max. T. [°C]:**	21.9	22.1	22	22.
		**Min. T. [°C]:**	21.8	21.9	21.9	21.9
		**Δ T [°C]:**	0.1	0.1	0.15	0.3
	**10 °C**	**Max. T. [°C]:**	10.1	10.2	11.1	10.3
		**Min. T. [°C]:**	10.1	10	10.6	10
		**Δ T [°C]:**	0.1	0.2	0.4	0.3
	**0 °C**	**Max. T. [°C]:**	1.4	1.7	1.7	1.3
		**Min. T. [°C]:**	0.9	1.2	1	0.5
		**Δ T [°C]:**	0.4	0.5	0.6	0.8

S 4. Test temperatures during cycling, indicated Δ T [°C] is calculated showing evolution during cycling.

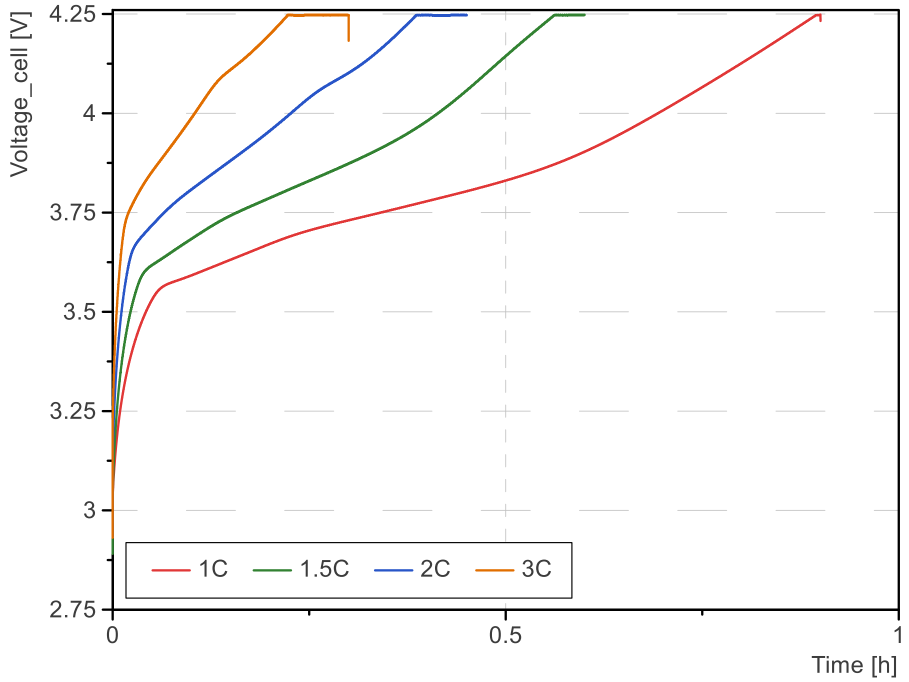

S 5. Comparison of charge cycle CC-phase duration at 22 °C (similar trend for all test temperatures).

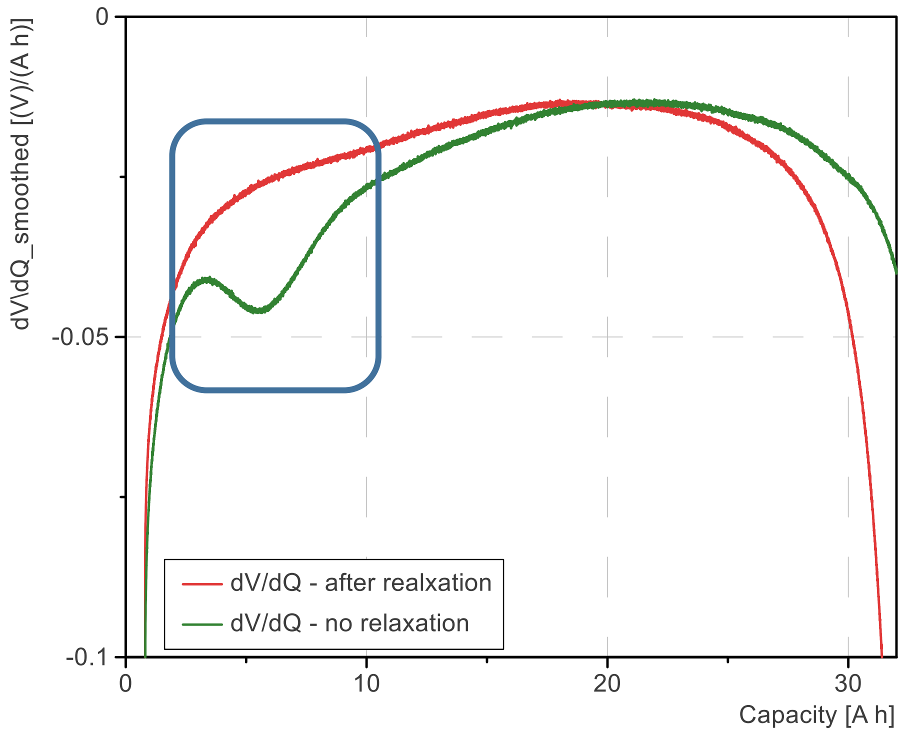

S 6: Effect of relaxation time on Li-stripping signal highlighted, for 10 °C at 1.5 C (same for all BCs).

S 7. Cell charge cycle data and capacity loss at 22 ºC.

Temperature 22 ºC									
**C-rate**	**Charging**						**DVA (Ah)**	**Q_{loss} (%)**	**$Q_{loss\ abs.}$ (Ah)**
	**Q_{cc} (Ah)**	**Q_{cv} (Ah)**	**Q_{total} (Ah)**	**End C-rate$_{(cv)}$**	**Time to 1C (s) in CV**	**ICA (Ah) peak 5**			
**1C**	57.7	0.53	58.23	0.87	0	0	0	0.3	0.19
**1.5C**	53.92	3.34	57.26	0.95	138	7.51	2	0.4	0.23
**2C**	46.84	6.49	54.95	0.90	232	16.53	5	2.2	1.21
**3**	39.53	12.67	52.2	1.35	343	15.83	7.37	4.7	2.48

S 8. Cell charge cycle data and capacity loss at 10 ºC.

Temperature 10 ºC									
**C-rate**	**Charging**						**DVA (Ah)**	**Q_{loss} (%)**	**$Q_{loss\ abs.}$ (Ah)**
	**Q_{cc} (Ah)**	**Q_{cv} (Ah)**	**Q_{total} (Ah)**	**End C-rate$_{(cv)}$**	**Time to 1C (s) in CV**	**ICA (Ah) peak 5**			
**1C**	52.80	4.09	56.89	0.57	0	0	0	0.21	0.12
**1.5C**	48.24	6.83	55.07	0.72	200	14.07	4.86	0.9	0.52
**2C**	33.79	15.02	48.81	0.68	496	15.95	9.87	3.7	1.85
**3C**	21.23	22.45	43.68	1.2	692	10.45	10	7.7	3.36

S 9. Cell charge cycle data and capacity loss at 0 ºC.

Temperature 0 ºC									
**C-rate**	**Charging**						**DVA (Ah)**	**Q_{loss} (%)**	**$Q_{loss\ abs.}$ (Ah)**
	**Q_{cc} (Ah)**	**Q_{cv} (Ah)**	**Q_{total} (Ah)**	**End C-rate$_{(cv)}$**	**Time to 1C (s) in CV**	**ICA (Ah) peak 5**			
**1C**	46.82	7.4	54.22	0.42	0	9.22	5	0.7	0.39
**1.5C**	32.14	17.06	49.2	0.63	429	13	8.7	3.7	1.84
**2C**	17.98	22.69	40.67	0.65	652	10.84	11.5	11.7	4.78
**3C**	10	24.22	34.22	0.93	812	6.15	10.49	15.2	5.23

S 10. Capacity stored during LP reaction peak 5.

Relative ICA 'peak 5' capacity (CC phase) (%)			
**C-rate**	**Temperature (°C)**		
	**22**	**10**	**0**
**1**	0	0	20
**1.5**	13.9	29.1	40
**2**	34.1	47.2	71.8
**3**	39.2	49.2	68.2

S 11. Relative ICA peak 5and DVA LP stripping capacities.

Relative DVA/ICA (%)			
**C-rate**	**Temperature (°C)**		
	**22**	**10**	**0**
**1**	0	0	54.22

**1.5**	26.6	34.5	66.9
**2**	30.2	61.8	106
**3**	46.5	95.69	170.5

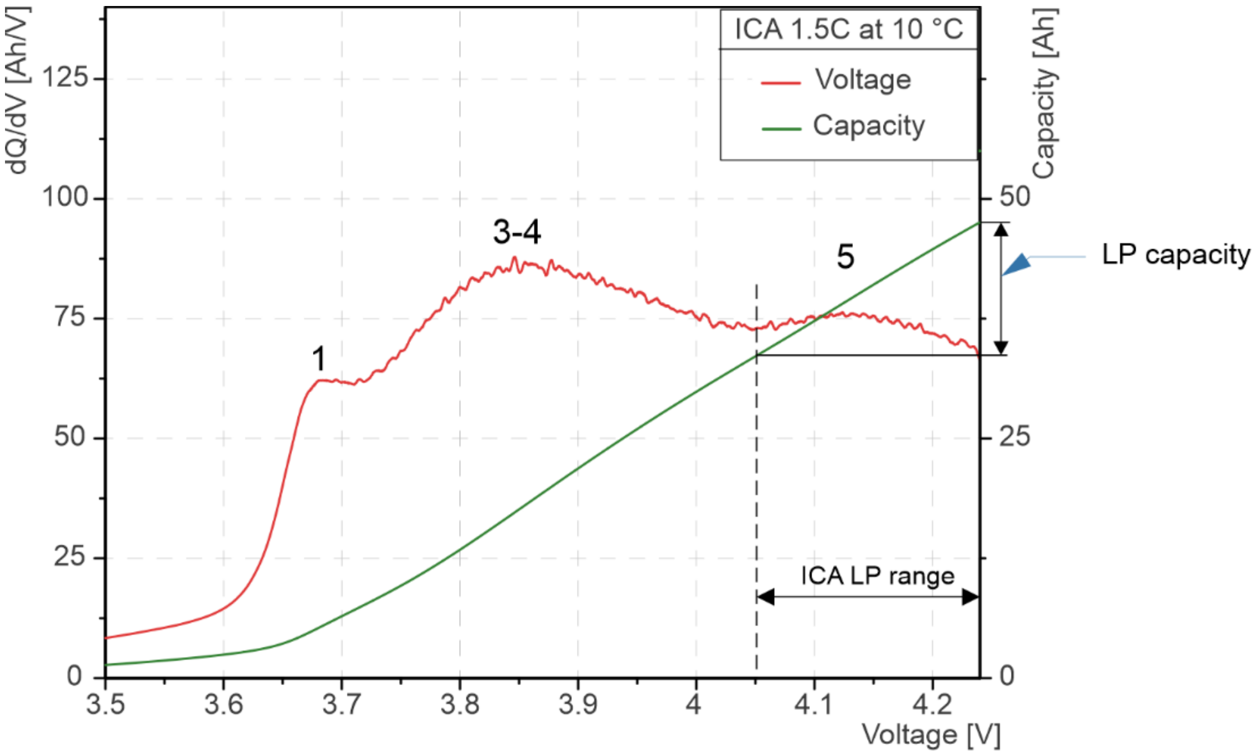

S 12. Illustration of ICA peak 5 LP capacity calculation in charge CC-phase.
